# Assessment of Salivary Polymerase Chain Reaction as a Non-invasive Method for Detecting Helicobacter pylori Infection in Patients With Symptomatic Gastritis

**DOI:** 10.7759/cureus.106091

**Published:** 2026-03-29

**Authors:** Mohd Shakir Khan, Minha Majeed Kak, Shahid Farooq, Seema Gupta

**Affiliations:** 1 Department of Oral Pathology, Shree Bankey Bihari Dental College and Research Centre, Ghaziabad, IND; 2 Department of Oral Pathology, Government Dental College and Hospital, Srinagar, IND; 3 Department of Dentistry, Government Medical College, Kathua, IND; 4 Department of Orthodontics, Kothiwal Dental College and Research Centre, Moradabad, IND

**Keywords:** biopsy, gastritis, helicobacter pylori, polymerase chain reaction, saliva

## Abstract

Introduction: *Helicobacter pylori *infection is a major etiological factor in chronic gastritis and several gastroduodenal diseases. Although gastric biopsy remains the gold standard for diagnosis, it is invasive and not always suitable for screening or repeated monitoring. Saliva-based polymerase chain reaction (PCR) has emerged as a promising non-invasive diagnostic approach. The present study aimed to evaluate the diagnostic performance of salivary PCR for detecting *H. pylori* infection and to compare its findings with gastric biopsy results in patients with symptomatic gastritis.

Materials and methods: This diagnostic accuracy study included 40 patients with symptomatic gastritis who were referred for upper gastrointestinal endoscopy. Unstimulated saliva samples were collected from all participants and analyzed using PCR targeting the 16S rRNA gene of *H. pylori*. Gastric biopsy with histopathological examination served as the reference standard. Diagnostic performance of salivary PCR was evaluated by calculating sensitivity, specificity, positive predictive value (PPV), negative predictive value (NPV), and overall accuracy. Statistical analysis included the Chi-square test and receiver operating characteristic (ROC) curve analysis.

Results: Salivary PCR detected *H. pylori* deoxyribonucleic acid in 33 of 40 patients. When compared with biopsy findings, salivary PCR demonstrated a sensitivity of 88.8% and a specificity of 75.0%. The PPV was 96.96%, while the NPV was 42.85%. The overall diagnostic accuracy was 87.5%. Although PCR positivity was higher among biopsy-confirmed cases, the association between salivary PCR and biopsy findings was not statistically significant (χ²=1.85, p=0.17). ROC analysis demonstrated good diagnostic performance, with an area under the curve (AUC) of 0.82.

Conclusion: Salivary PCR targeting the 16S rRNA gene demonstrates high sensitivity and good overall diagnostic accuracy for detecting *H. pylori* infection in patients with symptomatic gastritis. Although gastric biopsy remains the reference standard, salivary PCR represents a promising non-invasive adjunct that may be useful for screening and preliminary detection of *H. pylori* infection, particularly in settings where endoscopic evaluation is limited.

## Introduction

*Helicobacter pylori* is a gram-negative, microaerophilic, spiral-shaped bacterium that colonizes the gastric mucosa and is a major cause of chronic active gastritis, peptic ulcer disease, gastric adenocarcinoma, and mucosa-associated lymphoid tissue (MALT) lymphoma [1]. Classified as a class I carcinogen by the International Agency for Research on Cancer, it promotes malignancy through persistent inflammation, epithelial damage, and virulence factors such as cytotoxin-associated gene A (CagA) and vacuolating cytotoxin A (VacA) [2]. Infection is typically acquired in childhood and persists lifelong unless eradicated, with transmission occurring mainly via oral-oral and fecal-oral routes, particularly under conditions of poor hygiene and household crowding [1-3].

Increasing evidence points to the oral cavity, including saliva, dental plaque, and other oral sites, as a potential extra-gastric reservoir and possible source of transmission. *H. pylori *deoxyribonucleic acid (DNA) has been detected in the saliva of both symptomatic and asymptomatic individuals, suggesting the oral environment may serve as an important niche for the organism [4]. Conventional diagnosis depends primarily on invasive methods such as endoscopic biopsy with histopathology, rapid urease testing, or culture, approaches that are accurate but involve patient discomfort, procedural risks, and higher costs, limiting their use for screening or follow-up [5]. Non-invasive alternatives such as the urea breath test and stool antigen test perform well but have constraints in detecting low bacterial loads or confirming viable organisms at extra-gastric sites [5,6].

Polymerase chain reaction (PCR) targeting conserved genes such as 16S rRNA provides a sensitive, specific, and direct molecular detection method applicable to various specimens, including saliva. Saliva offers clear advantages: it is easy to collect, non-invasive, painless, cost-effective, and suitable for repeated sampling, making it promising for screening, epidemiological studies, and treatment monitoring [5,7].

The present study aimed to detect the presence of *H. pylori *in the saliva of patients with symptomatic gastritis using PCR targeting the 16S rRNA gene, and to compare the salivary PCR findings with endoscopic antral biopsy results. This comparison was performed to evaluate the potential of saliva as a non-invasive diagnostic specimen for *H. pylori* infection. The study assessed the presence of *H. pylori* DNA in saliva samples obtained from patients with clinically suspected gastritis using PCR and examined the agreement between salivary PCR results and histological detection of *H. pylori *in gastric biopsy specimens. Furthermore, the diagnostic performance of salivary PCR was evaluated by calculating sensitivity, specificity, positive predictive value (PPV), negative predictive value (NPV), and overall diagnostic accuracy relative to the biopsy-based reference method.

## Materials and methods

Study design and ethical approval

This diagnostic accuracy study was conducted in the Department of Oral Pathology, Kothiwal Dental College and Research Centre, Moradabad, India, from August 2021 to December 2022. The study protocol was reviewed and approved by the Institutional Ethical Review Board (KDCRC/IERB/12/2020/40 dated 26 December 2020). All procedures were carried out in accordance with the ethical principles outlined in the Declaration of Helsinki. Prior to participation, written informed consent was obtained from all individuals included in the study.

Study population and eligibility criteria

The study included patients presenting with clinical symptoms suggestive of gastritis who were referred for diagnostic upper gastrointestinal endoscopy. Symptoms included dyspepsia, epigastric pain, nausea, and abdominal discomfort. Only adult patients who provided written informed consent and were scheduled for endoscopic evaluation were included. Individuals who had received antibiotics, proton pump inhibitors, or H₂ receptor blockers within the previous two months were excluded to avoid interference with bacterial detection. Additional exclusion criteria included pregnancy, a history of upper gastrointestinal bleeding, and the presence of systemic illnesses that could influence the study outcomes.

Sample size estimation

The sample size was calculated based on previously reported diagnostic performance of salivary PCR for the detection of *H. pylori*. Assuming an expected sensitivity of approximately 80%, a precision margin of 15%, and a confidence level of 95%, the minimum required sample size was estimated to be 36 participants [8]. To compensate for potential dropouts or inadequate specimens, the final sample size was increased to 40 participants.

Sample collection

Gastric mucosal biopsy specimens were obtained from symptomatic patients during fiberoptic video gastroendoscopy performed under aseptic conditions by a qualified gastroenterologist. Two biopsy samples were collected from the antral region using sterile biopsy forceps. To minimize the risk of contamination, endoscopes and instruments were disinfected using 2% glutaraldehyde for 30 minutes before each procedure. One biopsy specimen was preserved in 10% buffered formalin for histopathological examination, while the other specimen was processed for molecular analysis, where required [9].

Unstimulated whole saliva samples were collected from all participants prior to endoscopy to avoid procedural influence. Saliva collection was performed between 7:00 AM and 8:00 AM using the spitting method. Participants were seated comfortably with their heads slightly tilted forward and were instructed to avoid speaking, swallowing, or excessive head movement during the collection period. After clearing the oral cavity of residual saliva, participants expectorated into a sterile graduated container at one-minute intervals for approximately 10 minutes. The collected saliva was immediately transferred into sterile containers containing digestion buffer composed of 100 mM NaCl, 10 mM Tris-HCl, 25 mM EDTA, and 1% SDS, and stored appropriately until DNA extraction.

Histopathological examination

Biopsy specimens fixed in 10% buffered formalin were processed using routine histopathological techniques. The tissues were dehydrated through graded alcohol concentrations (70%, 80%, 90%, and 100%), cleared in chloroform, and embedded in paraffin wax. Sections of approximately 4 μm thickness were cut using a semiautomatic microtome and mounted on egg albumin-coated slides. Following deparaffinization in xylene and rehydration through graded alcohols, the sections were stained using modified Giemsa stain. Under microscopic examination, *H. pylori *organisms were identified as curved or S-shaped purplish-blue bacilli measuring approximately 2-4 μm in length and 0.5-1 μm in thickness. Bacteria observed along the epithelial surface or within the mucosal layer were considered indicative of infection.

Deoxyribonucleic acid extraction from saliva

Genomic DNA was isolated from saliva samples using a commercial DNA extraction kit manufactured by Real Biotech Corporation, with minor modifications to the manufacturer’s protocol. Briefly, 300 µL of saliva was combined with 900 µL of red blood cell lysis buffer and incubated at room temperature for five minutes, followed by centrifugation. The supernatant was carefully removed, and the remaining pellet was resuspended again in red blood cell lysis buffer. Cellular lysis was achieved by the addition of guanidine-based buffer, followed by incubation at room temperature with intermittent mixing. Ethanol was subsequently added to facilitate the binding of DNA to the column membrane. The mixture was transferred to a genomic DNA binding column placed in a collection tube and centrifuged to allow attachment of DNA to the membrane. The column was then washed sequentially using appropriate wash buffers to remove residual impurities and subsequently dried by centrifugation. Finally, DNA was eluted using preheated elution buffer and stored at 4^°^C until PCR amplification.

Polymerase chain reaction

PCR was performed to amplify the 16S rRNA gene of *H. pylori*. The primer sequences used were forward primer 5′-TAAGAGATCAGCCTATGTCC-3′ and reverse primer 5′-TCCCACGCTTTAAGCGCAAT-3′, which produce an amplicon of 534 base pairs. PCR reactions were carried out using a commercially available master mix containing the necessary reagents for amplification. Thermal cycling conditions included an initial denaturation step at 95°C for five minutes, followed by 40 cycles consisting of denaturation at 94°C for 30 seconds, annealing at 56°C for 30 seconds, and extension at 72°C for one minute. A final extension step was performed at 72°C for five minutes. Positive control DNA from *H. pylori *(ATCC 26695) and a negative control without a DNA template were included in each run to validate the PCR procedure.

Gel electrophoresis and visualization

PCR products were analyzed using agarose gel electrophoresis. Amplified products were separated on a 2% agarose gel containing ethidium bromide and run at 120 V. The gels were visualized under ultraviolet transillumination, and the presence of a 534 base pair band corresponding to the positive control was considered indicative of the presence of *H. pylori* DNA. Gel documentation was performed to record the results.

Statistical analysis

Statistical analysis was performed using the statistical software (IBM SPSS Statistics, version 20.0, IBM Corp., Armonk, NY, USA). Continuous variables were expressed as mean and standard deviation, whereas categorical variables were presented as frequencies and percentages. The Shapiro-Wilk test was applied to evaluate the normality of continuous data. In this study, gastric biopsy histology served as the reference standard for the diagnosis of *H. pylori* infection. The diagnostic performance of salivary PCR was evaluated by calculating sensitivity, specificity, PPV, NPV, and overall diagnostic accuracy with a receiver operating characteristic (ROC) curve, each with corresponding 95% confidence intervals. The association between salivary PCR findings and biopsy results was assessed using the Chi-square test, and agreement between the two diagnostic methods was evaluated using Cohen’s Kappa coefficient. A two-tailed p-value of less than 0.05 was considered statistically significant.

## Results

A total of 40 patients with clinically suspected gastritis were included in the study. The mean age of the participants was 42.5±12.3 years, with an age range of 22 to 70 years. The study population consisted of 17 males (42.5%) and 23 females (57.5%), showing a slight female predominance. Most patients presented with dyspepsia, while epigastric pain was reported by 30 patients (75%). None of the participants were asymptomatic, reflecting the symptomatic nature of the study population. Regarding smoking status, 13 patients (32.5%) were smokers, whereas 27 patients (67.5%) were non-smokers. These findings indicate a predominantly middle-aged symptomatic population consistent with the clinical presentation of gastritis (Table 1).

**Table 1:** Baseline demographic and clinical characteristics of the study population Values are presented as mean±SD for continuous variables and as frequency (percentage) for categorical variables. SD: standard deviation

Parameter	Characteristic	Gastritis group (n=40)
Age (years)	Mean±SD	42.5±12.3
	Range	22-70
Sex, n (%)	Male	17 (42.5%)
	Female	23 (57.5%)
Clinical symptoms, n (%)	Dyspepsia	36 (90.0%)
	Epigastric pain	30 (75.0%)
	Asymptomatic	0 (0%)
Smoking status, n (%)	Smoker	13 (32.5%)
	Non-smoker	27 (67.5%)

The comparison between salivary PCR and gastric biopsy findings is shown in Table 2. Among the 40 patients evaluated, gastric biopsy detected *H. pylori *infection in 36 cases, while four cases were negative. Salivary PCR identified 32 true positive cases, three true negative cases, four false negative cases, and one false positive case when compared with biopsy results. Although PCR positivity was higher among biopsy-confirmed cases, the association between salivary PCR and biopsy findings did not reach statistical significance (χ²=1.85, p=0.17).

**Table 2:** Comparison of salivary PCR and gastric biopsy findings for the detection of Helicobacter pylori Data presented as frequency (percentage column, row). Gastric biopsy histopathology served as the reference standard for the diagnosis of *H. pylori* infection; p>0.05 indicates that the result is not statistically significant. PCR: polymerase chain reaction

Parameters		Gastric biopsy (gold standard)		Total	Chi stats	P-value
		Positive	Negative			
Saliva PCR	Positive	32 (88.8, 96.6)	1 (25.0, 3.4)	33 (82.5, 100.0)	1.85	0.17
	Negative	4 (11.2, 57.1)	3 (75.0, 42.9)	7 (17.5, 100.0)		
	Total	36 (100.0, 90.0)	4 (100.0, 10.0)	40 (100.0, 100.0)		

The diagnostic performance of salivary PCR for detecting *H. pylori *infection using gastric biopsy as the reference standard is presented in Table 3. Salivary PCR demonstrated a sensitivity of 88.8% (32/36) and a specificity of 75.0% (3/4). The PPV was 96.96%, indicating that most PCR-positive cases were correctly identified. However, the NPV was 42.85%, suggesting limited ability of the test to rule out infection in PCR-negative cases. The overall diagnostic accuracy of salivary PCR was 87.5%, reflecting good agreement with the reference biopsy method. However, the relatively small number of biopsy-negative cases resulted in wider confidence intervals for specificity and NPV.

**Table 3:** Diagnostic performance of salivary PCR for detection of Helicobacter pylori Diagnostic performance metrics were calculated using gastric biopsy as the reference standard. Sensitivity, specificity, predictive values, and accuracy are expressed as percentages. PPV: positive predictive value; NPV: negative predictive value; AUC: area under the curve

Diagnostic parameter	Value	95% confidence interval
Sensitivity	88.8%	73.9%-96.9%
Specificity	75.0%	19.4%-99.4%
PPV	96.96%	84.2%-99.9%
NPV	42.85%	9.9%-81.6%
Accuracy	87.50%	73.2%-95.8%
AUC	0.82	-0.44% to 2.08%

**Figure 1:**
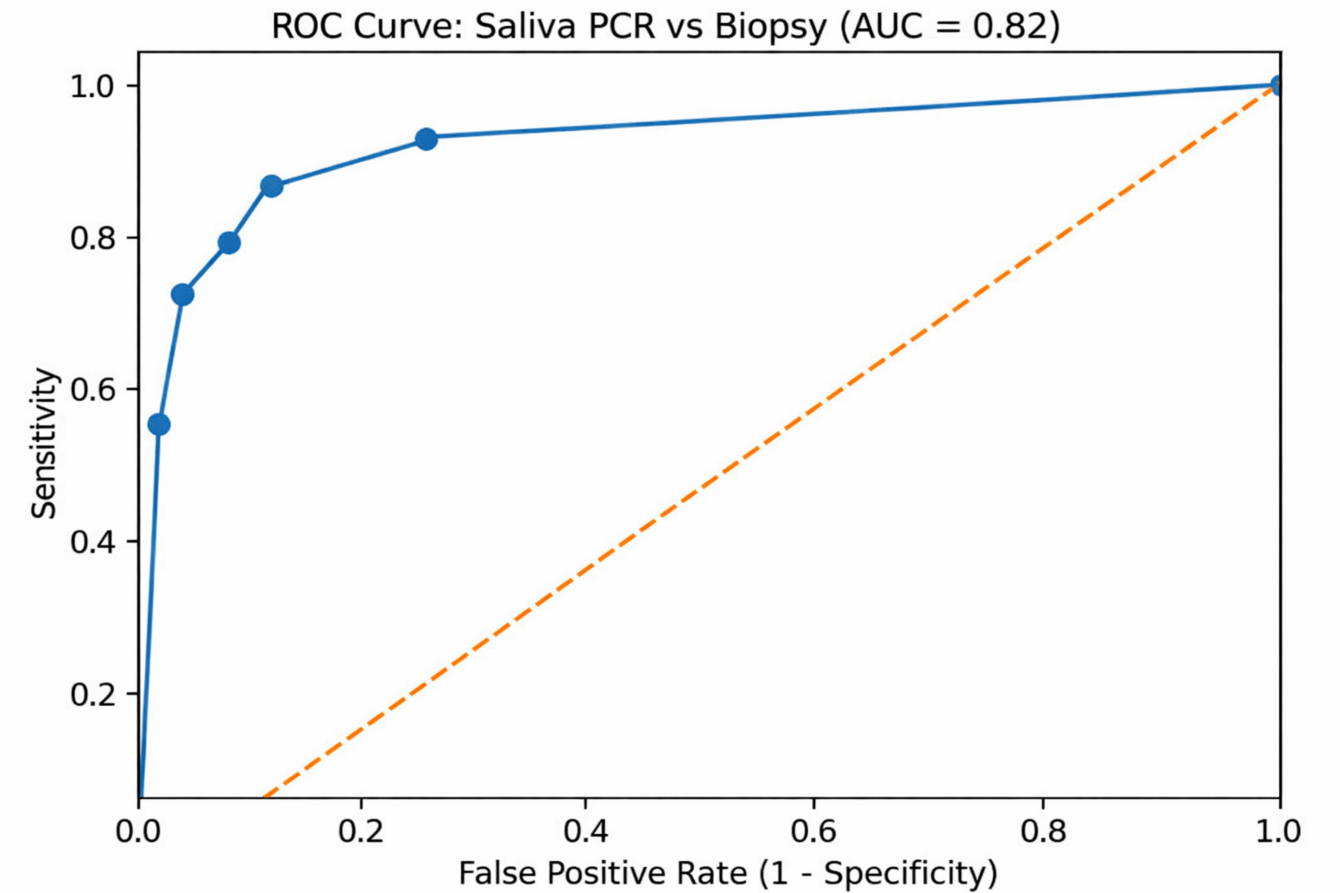
ROC curve for the diagnostic accuracy of the salivary PCR method for detecting Helicobacter pylori ROC: receiver operating characteristic; PCR: polymerase chain reaction;* *AUC: area under the curve

ROC curve analysis was performed to evaluate the overall diagnostic performance of salivary PCR. The area under the curve (AUC) was 0.82, indicating good discriminatory ability of salivary PCR in distinguishing *H. pylori*-positive and negative cases (Figure 1). The ROC curve lies well above the reference line, further supporting the diagnostic potential of salivary PCR as a non-invasive method for detecting *H. pylori* infection.

## Discussion

The present study evaluated the diagnostic utility of salivary PCR for detecting *H. pylori* infection in patients with symptomatic gastritis and compared the findings with gastric biopsy histology, which served as the reference standard. The results demonstrated good diagnostic performance of salivary PCR, with a sensitivity of 88.8%, specificity of 75.0%, and an overall diagnostic accuracy of 87.5%. ROC analysis further confirmed good discriminatory ability, with an AUC of 0.82. These findings support the potential of saliva as a reliable and non-invasive specimen for detecting *H. pylori* infection.

The high sensitivity observed in the present study is consistent with previous investigations reporting sensitivities ranging from 80% to 95% when PCR targets conserved genes such as 16S rRNA [10,11]. The use of 16S rRNA primers likely contributed to this high detection rate because this gene is highly conserved and present in multiple copies, increasing the likelihood of detecting bacterial DNA even when the bacterial load is low. Mapstone et al. [10] were among the first to demonstrate the presence of *H. pylori* DNA in saliva and dental plaque using PCR, suggesting that the oral cavity may serve as a reservoir for the organism. Subsequent studies by Song et al. [11] and Momtaz et al. [12] further supported these findings by demonstrating concordance between oral and gastric detection of *H. pylori*.

The specificity observed in the present study was lower than the sensitivity, but it remains clinically acceptable for a screening method. False-positive PCR results may arise from transient bacterial DNA present in the oral cavity, contamination due to gastroesophageal reflux, or the detection of non-viable bacterial DNA fragments. Because PCR detects genetic material irrespective of bacterial viability, it may identify residual DNA even in the absence of active gastric colonization. This limitation has been acknowledged in previous studies [13]. Zou and Li [13] conducted a meta-analysis demonstrating a significant association between the presence of *H. pylori* in the oral cavity and gastric mucosa, supporting the concept that the oral cavity may act as a potential reservoir and possible source of gastric reinfection.

In the present study, four biopsy-positive cases were negative on salivary PCR. These false-negative results may be explained by low salivary bacterial load, uneven bacterial distribution in the oral cavity, or the presence of inhibitory substances in saliva that interfere with PCR amplification. Saliva contains various enzymes and host-derived components that may reduce PCR efficiency if not adequately removed during DNA extraction. Despite these limitations, the overall diagnostic accuracy observed in this study suggests that salivary PCR can correctly identify the majority of infected individuals.

The ROC curve analysis demonstrated good diagnostic performance of salivary PCR, with an AUC of 0.82, indicating reliable discrimination between infected and non-infected individuals. Although gastric biopsy remains the gold standard for diagnosing *H. pylori* infection, its invasive nature limits its use for screening or repeated monitoring. In contrast, saliva collection is simple, painless, and inexpensive, making it particularly suitable for large-scale screening or follow-up evaluation in resource-limited settings. The diagnostic performance observed in the present study is comparable with other non-invasive diagnostic methods, such as the urea breath test, which has reported sensitivities and specificities exceeding 90% [14].

Previous studies have also compared PCR with histological detection of *H. pylori*. Moalla et al. [15] evaluated the performance of histology compared with PCR in symptomatic patients and reported that PCR demonstrated higher sensitivity, particularly in cases with low bacterial density or previous eradication therapy, where histology may underestimate infection. Additionally, Sekhar Goud et al. [8] demonstrated successful detection of *H. pylori* DNA in saliva using PCR and suggested that saliva may represent a useful non-invasive specimen for identifying infection.

The oral cavity comprises multiple distinct ecological niches, including saliva, supragingival plaque, subgingival plaque, and the dorsum of the tongue, each characterized by unique microbial compositions, oxygen gradients, and biofilm structures. These sites differ significantly in their ability to support bacterial colonization and persistence. While saliva represents a transient and diluted microbial environment, structured biofilm niches such as dental plaque and the tongue dorsum may provide more favorable conditions for sustained colonization of *H. pylori*. Therefore, the detection of *H. pylori* in saliva may not necessarily reflect stable colonization, highlighting the importance of considering site-specific differences when interpreting oral detection of the organism.

Clinical implications

From a clinical perspective, salivary PCR offers several advantages. It is non-invasive, painless, and easily repeatable, making it suitable for screening large populations and monitoring treatment response. In settings where endoscopic facilities are limited, salivary PCR may serve as an adjunctive or preliminary diagnostic method for detecting *H. pylori *infection.

Limitations

Despite the encouraging findings, certain limitations should be acknowledged. First, the sample size was relatively small and derived from a single center, which may limit the generalizability of the results. Second, PCR detects bacterial DNA but does not confirm bacterial viability and therefore may identify non-viable organisms. Third, key oral health-related variables, including oral hygiene status, periodontal disease, number of remaining teeth, use of antiseptic mouthwashes such as chlorhexidine, and recent dental treatment, were not systematically recorded or controlled. These factors can significantly influence the oral microbial load and may affect the detection of *H. pylori*, potentially introducing variability or bias in salivary PCR results. Additionally, the use of saliva as the sole sampling medium may not adequately reflect site-specific colonization within structured oral niches such as subgingival plaque or the tongue dorsum. Moreover, variations in salivary flow rate and oral hygiene practices may dilute bacterial DNA in saliva, potentially reducing concentrations below the PCR detection threshold and contributing to false-negative results. Future multicenter studies with larger cohorts, comprehensive periodontal assessment, and site-specific sampling combined with quantitative PCR are warranted to better elucidate the role of the oral cavity in *H. pylori* colonization and improve diagnostic reliability.

## Conclusions

Within the limitations of this single-center study, salivary PCR targeting the 16S rRNA gene demonstrated high sensitivity for the detection of *H. pylori* in patients with symptomatic gastritis. However, the relatively low negative predictive value, wide variability in specificity estimates due to the small number of biopsy-negative cases, and the non-significant statistical association warrant cautious interpretation of the findings. Salivary PCR may be considered a useful non-invasive adjunctive tool for preliminary screening, particularly in settings where endoscopic evaluation is limited, but it cannot replace established diagnostic methods such as gastric biopsy. Further large-scale, multicenter, and longitudinal studies incorporating detailed oral health assessment and site-specific sampling are required to validate these findings and to better define the role of the oral cavity as a potential reservoir for *H. pylori*, as well as to evaluate changes in salivary PCR status following eradication therapy.

## References

[1] Reyes VE (2023). Helicobacter pylori and its role in gastric cancer. Microorganisms.

[2] Ansari S, Ahmed N (2025). Pathogenicity of Helicobacter pylori-associated gastric cancer. World J Clin Oncol.

[3] Miller AK, Williams SM (2021). Helicobacter pylori infection causes both protective and deleterious effects in human health and disease. Genes Immun.

[4] Costa LC, Carvalho MD, Vale FF, Marques AT, Rasmussen LT, Chen T, Barros-Pinheiro M (2024). Helicobacter pylori in oral cavity: current knowledge. Clin Exp Med.

[5] Costa LC, Das Graças Carvalho M, La Guárdia Custódio Pereira AC, Teixeira Neto RG, Andrade Figueiredo LC, Barros-Pinheiro M (2024). Diagnostic methods for Helicobacter pylori. Med Princ Pract.

[6] Sabbagh P, Mohammadnia-Afrouzi M, Javanian M (2019). Diagnostic methods for Helicobacter pylori infection: ideals, options, and limitations. Eur J Clin Microbiol Infect Dis.

[7] Cardos AI, Maghiar A, Zaha DC, Pop O, Fritea L, Miere Groza F, Cavalu S (2022). Evolution of diagnostic methods for Helicobacter pylori infections: from traditional tests to high technology, advanced sensitivity and discrimination tools. Diagnostics (Basel).

[8] Sekhar Goud EV, Kannan R, Rao UK, Joshua E, Tavaraja R, Jain Y (2019). Identification of Helicobacter pylori in saliva of patients with and without gastritis by polymerase chain reaction. J Pharm Bioallied Sci.

[9] Nair S, Wasnik N, Bhole SS (2025). A clinicopathological study of the correlation between H. pylori and dyspepsia: a cross-sectional study. Cureus.

[10] Mapstone NP, Lynch DA, Lewis FA, Axon AT, Tompkins DS, Dixon MF, Quirke P (1993). Identification of Helicobacter pylori DNA in the mouths and stomachs of patients with gastritis using PCR. J Clin Pathol.

[11] Song Q, Haller B, Schmid RM, Adler G, Bode G (1999). Helicobacter pylori in dental plaque: a comparison of different PCR primer sets. Dig Dis Sci.

[12] Momtaz H, Souod N, Dabiri H, Sarshar M (2012). Study of Helicobacter pylori genotype status in saliva, dental plaques, stool and gastric biopsy samples. World J Gastroenterol.

[13] Zou QH, Li RQ (2011). Helicobacter pylori in the oral cavity and gastric mucosa: a meta-analysis. J Oral Pathol Med.

[14] Logan RP, Walker MM (2001). ABC of the upper gastrointestinal tract: epidemiology and diagnosis of Helicobacter pylori infection. Br Med J.

[15] Moalla M, Chtourou L, Mnif B (2024). Assessment of histology's performance compared with PCR in the diagnosis of Helicobacter pylori infection. Future Sci OA.

